# Remineralization Strategies in Oral Hygiene: A Position Paper of Italian Society of Oral Hygiene Sciences

**DOI:** 10.2174/1874210601812010011

**Published:** 2018-01-17

**Authors:** Consuelo Sanavia, Marco Tatullo, Jessica Bassignani, Silvia Cotellessa, Giulia Fantozzi, Giovanna Acito, Alessia Iommiello, Lorella Chiavistelli, Silvia Sabatini, Gianna Maria Nardi

**Affiliations:** 1 Italian Society of Oral Hygiene Sciences-S.I.S.I.O. working group, Pisa, Italy; 2 Calabrodental SRL, Crotone, Italy; 3 Department of Oral and Maxillofacial Science, University of Rome “Sapienza”, Rome, Italy

The correct list of authors’ names is provided and replaced online which is mentioned as under:

***Consuelo Sanavia*** ^1^ ^#^ ^*^ ***Marco Tatullo*** ^2^ ^#^ ***Jessica Bassignani*** ^1^ ***Silvia Cotellessa*** ^1^ ***Giulia Fantozzi*** ^1^ ***Giovanna Acito*** ^1^ ***Alessia Iommiello*** ^1^ ***Lorella Chiavistelli*** ^1^ ***Silvia Sabatini*** ^1^ ***Gianna Maria Nardi*** ^1^ ^3^

The published list of authors was: 

***Consuelo Sanavia*** ^1^ *^,^* ^#^ *^,^* ^*^ ***, Marco Tatullo*** ^2^ *^,^* ^#^ ***, Jessica Bassignani*** ^1^ ***, Silvia Cotellessa*** ^1^ ***, Giulia Fantozzi*** ^1^ ***, Giovanna Acito*** ^1^ ***, Alessia Iommiello*** ^1^ ***Lorella Chiavistelli*** ^1^ ***Alessia Iommiello*** ^1^ ***Silvia Sabatini*** ^1^ ***Gianna Maria Nardi*** ^1^*^^* ^3^

